# Confronting mortality: A meta-analysis and systematic review of psychedelic experiences and death anxiety

**DOI:** 10.1177/02698811261424199

**Published:** 2026-03-09

**Authors:** Alicia Cohorst, Petri J. Kajonius

**Affiliations:** 1 Department of Psychology, Lund University, Sweden; 2 Department of Clinical Sciences, Lund University, Sweden

**Keywords:** psychedelics, death anxiety, existential distress, fear of death, death acceptance

## Abstract

This meta-analysis and systematic review examined the effects of psychedelic substances on death anxiety, specifically evaluating whether psychedelic experiences are associated with statistically significant reductions in death anxiety. A systematic search identified 8 studies suitable for meta-analysis and 10 additional studies for systematic review. Using a random-effects model, the meta-analysis revealed a significant overall effect (Cohen’s *d* = 0.70; 95% confidence interval [0.42, 0.97]), indicating a moderate-to-large reduction in death anxiety following psychedelic administration. Subgroup analyses showed stronger effects in clinically controlled settings compared to the general population. A mixed-effects meta-regression also indicated that particularly mystical experiences were positively associated with reductions in death anxiety (*B* = 4.90, *p* = 0.050). Moderator effects by substance type were not significant. The qualitative review reaffirmed these results and identified themes of ego dissolution, emotional catharsis, and living in the present moment. Psychedelic-assisted interventions may be effective for reducing death anxiety, particularly in end-of-life care. However, limitations include a small number of studies, potential publication bias, and high heterogeneity in outcome measurement. Future research should employ more methodologically rigorous trials in order to clarify the mechanisms of psychedelic-assisted approaches to death anxiety.

## Introduction

In recent years, there has been growing interest in the therapeutic potential of psychedelics, including in addressing existential concerns such as death anxiety. Preliminary clinical trials and qualitative reports suggest that certain psychedelic experiences may significantly reduce death anxiety, offering therapeutic potential for end-of-life care and existential psychotherapy. To better understand the scope and consistency of this effect, this study objective is to conduct a meta-analysis and systematic review of the existing literature on psychedelics and death anxiety, synthesizing both quantitative and qualitative outcomes.

A scoping review was recently conducted by Barr et al. (2025) examining serotonergic psychedelics and attitudes toward death. While this review provided a valuable synthesis, its scope was intentionally wide, focusing on all attitudinal change rather than quantifying specific effects on death anxiety or identifying moderators of this relationship. The present study differs in scope and methodology, focusing specifically on death anxiety and employing a quantitative meta-analytic approach with stricter inclusion criteria to assess effect sizes and potential moderators, supplemented by a systematic review of qualitative and mixed-method evidence to contextualize quantitative findings.

This approach aims to clarify whether psychedelics reliably reduce death anxiety, how effects differ across clinical vs. non-clinical populations, and whether mystical experiences may moderate outcomes. In doing so, this review contributes a focused, data-driven synthesis that complements existing narrative reviews and helps clarify where therapeutic potential exists and where further research is needed.

### Death anxiety

Death anxiety, also known as thanatophobia, refers to the apprehension and fear associated with the awareness of one’s mortality. It is defined as a psychological state of distress characterized by a dread of annihilation and the loss of existence (Carpenito-Moyet, 2008). Unlike general anxiety, which may be directed at concrete or situational threats, death anxiety arises from a uniquely existential fear and the inability to fully comprehend what it means to cease existing. According to Becker’s (1973) *The Denial of Death*, much of human behavior is motivated by an unconscious attempt to deny or repress the awareness of mortality. Terror Management Theory extends this framework by proposing that awareness of death creates the potential for debilitating terror, which is buffered through psychological defenses such as cultural worldviews, self-esteem, and close relationships that provide a sense of “symbolic immortality” (Greenberg et al., 1986). Empirical research shows that subtle mortality reminders, or mortality salience inductions, increase worldview defense, self-esteem maintenance, and bias toward those who uphold one’s cultural values (Greenberg et al., 1986; Rosenblatt et al., 1989). These findings suggest that much of human motivation and behavior operates, at least in part, to mitigate death-related anxiety by preserving a sense of meaning and symbolic immortality.

Cognitions associated with death anxiety often encompass thoughts about dying prematurely, the state of being dead, fear of the unknown, concerns about the impact of one’s death on loved ones, and existential reflections on the cessation of the self (Blomstrom et al., 2022). Although death anxiety may not always be consciously accessible, individuals engage in self-regulatory strategies such as inhibition and distraction in order to suppress death-related thoughts (Lehto and Stein, 2009). However, over time, this regulation may fail, leading to heightened or chronic death-related distress (Gaillot et al., 2006).

Given its pervasive role across a range of psychopathologies, death anxiety can be conceptualized as a transdiagnostic construct (Iverach et al., 2014). Similar to other transdiagnostic constructs like perfectionism and rumination, death anxiety may operate as a shared mechanism that drives maladaptive thought patterns and avoidance behaviors across diagnostic boundaries. A strong association has been found between death-related fears and clinical symptoms in disorders such as panic disorder, OCD, illness anxiety disorder, and borderline personality disorder (Iverach et al., 2014; Menzies et al., 2025). As with other transdiagnostic constructs, interventions that directly address death anxiety may lead to broader symptom reduction, increase treatment efficiency, and improve long-term outcomes (Iverach et al., 2014).

Age-related differences in death anxiety show a non-linear trajectory, with anxiety peaking in the twenties and later declining, although women experience a unique secondary spike in their fifties not seen in men (Russac et al., 2007). This suggests that developmental and gender-specific factors may underlie these patterns. In addition, women generally report higher levels of death anxiety than men, though this difference attenuates by late adulthood (Russac et al., 2007). Religiosity has also been investigated as a potential factor influencing death anxiety, with meta-analytic evidence indicating a curvilinear association: individuals who are either strongly religious or strongly non-religious report lower death anxiety than those with more ambivalent or uncertain beliefs (Jong et al., 2018).

The World Health Organization and public health policies across nations have increasingly recognized the importance of addressing the mental health and quality of life of people with life-threatening diseases. Death anxiety, which can substantially influence these domains, is therefore not just a personal issue but a global public health concern (Li et al., 2025).

### Treatments and interventions

Despite prevalence and impact, death anxiety remains difficult to treat directly. Standard therapeutic interventions such as cognitive-behavioral therapy or mindfulness practices can be effective to some extent. A recent meta-analysis of psychosocial interventions found that treatments generally produced small-to-moderate reductions in death anxiety, with cognitive-behavioral approaches being particularly effective (Menzies et al., 2018). However, the review also highlighted substantial methodological limitations in the literature, including small sample sizes, heterogeneity of measures, and high risk of bias. Additionally, a phase I trial of an online Cognitive Behavioral Therapy (CBT) program specifically targeting death anxiety provided preliminary evidence for the feasibility and safety of scalable interventions, with the majority of participants showing clinically reliable reductions in death-related fears (Menzies et al., 2023).

A systematic review by Blomstrom et al. (2022) explores a range of therapeutic approaches in addressing thanatophobia, including psychotherapy, mindfulness, virtual reality, and psychedelic therapy, and concludes that while several interventions have potential for reducing death anxiety, none stand out as clearly superior. Each approach offers unique benefits and faces its own practical and therapeutic limitations.

Psychotherapy has shown potential in reducing death anxiety, particularly among individuals with terminal illnesses. There are several interventions that aim to help patients construct meaning and purpose in the face of mortality. One such intervention is Meaning-Centered Psychotherapy, originally developed for advanced-stage cancer patients to enhance their sense of meaning, peace, and purpose (Frankl, 1984). A condensed version focuses on identifying personal meaning and legacy in just three sessions, though results have varied across individuals (Rosenfeld et al., 2017).

Mindfulness-based interventions have demonstrated potential in alleviating psychological symptoms commonly associated with death anxiety, such as depression, anxiety, and distress. For example, a mindfulness-based psychoeducational intervention improved depression and quality of life in patients with chronic heart failure (Sullivan et al., 2009), while a tailored mindfulness program enhanced quality of life and acceptance among individuals with amyotrophic lateral sclerosis (Pagnini et al., 2017). Mindfulness has also been associated with reduced distress and anxiety in cancer patients (Carlson et al., 2004; Piet et al., 2012). These techniques may be especially useful in populations facing high mortality risk, as they can reduce emotional reactivity and increase acceptance of one’s condition and mortality. Overall, mindfulness interventions show consistent potential for improving psychological outcomes in populations likely to be impacted by death anxiety. However, many mindfulness studies are limited by small sample sizes, homogeneous samples, and the absence of control groups (Blomstrom et al., 2022).

Emerging research suggests that virtual reality (VR) technology can potentially explore and reduce death anxiety through immersive simulations. By placing individuals in controlled, first-person virtual environments, researchers can create embodied experiences that mirror aspects of dying, including dissociation from the physical body and encounters with symbolic death-related phenomena (Barberia et al., 2018; Bourdin et al., 2017; Glowacki 2024). Experiences in multiplayer VR settings have shown psychometric outcomes comparable to psychedelics, such as feelings of unity, ego-dissolution, and significant reductions in death anxiety (Barberia et al., 2018; Bourdin et al., 2017).

### Psychedelics

Psychedelics are a broad class of psychoactive substances that act as agonists of perception, cognition, and affect through their interactions with various neurotransmitter systems (Nichols, 2016). Classic psychedelics, such as psilocybin, lysergic acid diethylamide (LSD), and dimethyltryptamine (DMT), primarily exert their effects as agonists or partial agonists at the serotonin 2A receptor (5-HT2AR) (Nichols, 2016). Non-serotonergic compounds, such as 3,4-methylenedioxymethamphetamine (MDMA) and ketamine, produce psychedelic-like experiences via different primary mechanisms, including serotonin release and N-methyl-D-aspartate receptor antagonism (Johnson et al., 2019).

Following early findings in the 1950s and 1960s that linked psychedelics to improvements in mood disorders, alcohol dependence, and existential distress, research on psychedelics was largely halted by restrictive drug legislation (Carhart-Harris and Goodwin, 2017). In recent decades, however, a resurgence of interest has reestablished psychedelics as a potential therapeutic tool within psychiatry, particularly for conditions that are resistant to conventional treatments. Clinical trials have demonstrated that psilocybin and related compounds can produce rapid and sustained reductions in depressive and anxiety symptoms, often accompanied by experiences described as deeply meaningful or transformative (Griffiths et al., 2016; Ross et al., 2016). These findings have prompted renewed discussion about the medical and ethical implications of psychedelic therapy, including its potential role in addressing existential suffering and death anxiety among individuals facing terminal illness.

Contemporary research on psychedelics can broadly be divided into clinical and naturalistic studies. Clinical studies involve controlled administration of psychedelics within a therapeutic or medical setting, often accompanied by structured psychological support and standardized outcome measures. These trials aim to evaluate safety, efficacy, and mechanisms of therapeutic change, offering evidence for the medical use of psychedelics in treating depression, anxiety, and existential distress related to terminal illness (Griffiths et al., 2016; Grob et al., 2011; Ross et al., 2016). In contrast, naturalistic studies examine psychedelic experiences as they occur outside laboratory or clinical contexts, including recreational use. Although such studies lack experimental control, they provide insights into the enduring psychological and existential effects of psychedelics under real-world conditions and across diverse populations (Garcia et al., 2025; Yaden et al., 2017). Integrating findings from both approaches allows for a more comprehensive understanding of how psychedelic experiences may influence death anxiety and related constructs across settings.

Rather than solely reducing symptoms, psychedelic experiences seem to offer the potential for psychological insight, spiritual connection, and meaning-making; dimensions that may often be neglected in mainstream psychiatric treatment. For example, a recent self-reported survey found that 58% of psychedelic users ranked their experience among the top five most meaningful events in their lives, and 85% among the top ten (Kajonius et al., 2024). Ninety-four percent of users reported positive life improvements. Despite a likely self-selected sample, such results reinforce the idea that psychedelics may have significant and lasting effects on overall personal meaning and psychological growth, including how to related to one’s own death.

#### Mystical experience

Mystical-type experiences have often been described as among the most transformative effects induced by psychedelics. Such experiences have been framed by characteristics such as feelings of unity, sacredness, transcendence of time and space, ineffability, and a deep sense of meaning or truth (Stace, 1960). In modern research contexts, psilocybin and other classic psychedelics have been shown to reliably induce these experiences under controlled conditions (Griffiths et al., 2006, 2008, 2011). These mystical-type experiences have been linked to sustained improvements in psychological well-being, personal meaning, and life satisfaction, suggesting that the therapeutic potential of psychedelics may be closely tied to the depth of the mystical experience itself (Griffiths et al., 2011). In the context of death anxiety and existential distress, such experiences may offer a shift in perspective by diminishing fear, enhancing acceptance, and fostering a sense of continuity or connection beyond the self (MacLean et al., 2012).

Findings from Exline et al. (2023) show that people tend to frame “mystical” psychedelic experiences primarily through a spiritual lens, noting feelings of connection, divine love, and transcendence, rather than as signs of mental illness or solely neurobiological events. This interpretive framing can profoundly influence its emotional and existential impact. When such experiences are understood as encounters with a greater reality or higher consciousness, they may prompt lasting shifts in worldview.

Carhart-Harris and Friston (2019) argue that philosophically, mystical or spiritual insights arising from psychedelic experiences can be viewed as *psychologically real* even if their metaphysical truth remains uncertain. Such experiences may function as adaptive interpretations that help individuals make sense of profound or ambiguous states, effectively reducing psychological uncertainty. Psychedelic experiences may temporarily relax rigid top-down models, allowing new interpretations to emerge that, if integrated effectively, can lead to more flexible and adaptive worldviews (Carhart-Harris and Friston, 2019).

The rapid and sustained improvements in anxiety, depression, and quality of life reported in recent studies suggest that psychedelics could fill a critical gap in end-of-life care, particularly when integrated into therapeutic frameworks that support emotional processing and existential reflection. The effects observed in these trials are often described as spiritually significant or mystical, pointing to the possibility that therapeutic efficacy may not stem solely from neurochemical changes but also from the profound nature of the experiences themselves (Schutt et al., 2025). Together, these perspectives suggest that mystical experiences may facilitate psychological transformation not only through pharmacological effects, but by restructuring the individual’s cognitive and existential framework surrounding death.

### Study aim

Despite growing interest in the therapeutic potential of psychedelics, their effects on death anxiety remain understudied. As Li et al. (2025) argue, death anxiety affects survival quality, mental health, and care outcomes, and warrants more rigorous research attention. Given its growing recognition and clinical importance, death anxiety is a topic of central relevance in both research and practice. Psychological and existential dimensions such as meaning, unfinished business, and fear of non-existence are essential components of comprehensive care. Understanding and addressing death anxiety can lead to better end-of-life outcomes not only for patients, but also for their families (Li et al., 2025). Psychedelics, particularly when accompanied by mystical-type or emotionally significant experiences, may offer a novel approach to alleviating death-related distress.

Although multiple studies have examined this relationship, the evidence base is highly heterogeneous. Differences in substances, study designs, outcome measures, and sample characteristics make it difficult to draw overarching conclusions. A formal meta-analysis is therefore needed to synthesize the quantitative findings, evaluate the robustness of observed effects, and explore potential moderators such as substance type, clinical population, or intensity of mystical experience. Furthermore, a narrative synthesis can provide important context, especially for studies that use qualitative or retrospective approaches.

The aim of this study was to meta-analyze and systematically review the existing literature on the relationship between psychedelic use and death anxiety. Specifically, the study aimed to quantify the overall effect of psychedelics on death anxiety across studies, examine whether this effect varies depending on study characteristics such as sample type (clinical vs. non-clinical), type of psychedelic used, and methodological design, and explore whether the intensity of mystical experience moderates the relationship between psychedelic use and reductions in death anxiety.

This study is, to our knowledge, the first meta-analysis to systematically investigate the effects of psychedelic experiences on death anxiety across both clinical and non-clinical populations. Previous meta-analyses and reviews have primarily focused on clinical samples of patients facing life-threatening illnesses (Amaev et al., 2025; Bader et al., 2024; Jing et al., 2023; Marchi et al., 2024; Reiche et al., 2018; Schimmers et al., 2022; Schipper et al., 2022). While these studies are crucial, they reflect a limited subset of individuals experiencing death anxiety, which is not exclusive to those facing terminal diagnoses. A growing number of people in the general population are turning to psychedelics for personal growth, meaning-making, and psychological well-being, often in non-clinical contexts.

## Methods

### Study design

This study employed a meta-analytic approach to quantitatively synthesize the effects of psychedelic substances on death-related psychological outcomes. A systematic review was conducted in addition to the meta-analysis in order to include studies with relevant outcomes such as qualitative data or noncomparable quantitative data. Both the meta-analysis and systematic review were conducted in accordance with The Preferred Reporting Items for Systematic reviews and Meta-Analyses (PRISMA) guidelines (Page et al., 2021), and the procedure included systematic study selection, effect size extraction, and statistical synthesis using a random-effects model. Moderator analyses were conducted to explore the potential influence of substance type on effect size. The dependent variable in all analyses was the change in death-related psychological outcomes following psychedelic use.

### Eligibility criteria

Studies were included if they involved administration/consumption of a psychedelic substance (e.g. psilocybin, LSD, DMT, MDMA), measured death anxiety and/or death acceptance using a validated psychometric instrument at any time post-consumption, reported sufficient data to calculate an effect size and a measure of variability, and were peer-reviewed and published in English. In order to be included in the meta-analysis, studies were required to employ a within-subject (pre-post) design. Studies using between-subject comparisons were included in the systematic review instead. Both experimental and quasi-experimental designs were eligible. Studies were excluded if they lacked sufficient statistical information, assessed death anxiety using single or non-validated measures, measured unrelated constructs, or involved overlapping samples with other included studies. Studies with outcome measures such as general anxiety or depression associated with life-threatening illnesses were excluded unless they specifically assessed death anxiety, fear of death, or death acceptance.

### Measuring death anxiety

The Collett-Lester Fear of Death Scale – Revised (Lester and Abdel-Khalek, 2003) is a 28-item measure assessing nuanced dimensions of death anxiety. It includes four subscales, each with seven items: Death of Self, Dying of Self, Death of Others, and Dying of Others. Items are rated on a 6-point Likert scale, allowing for greater sensitivity to individual differences in fear and concern across these domains. The scale has demonstrated excellent internal consistency across studies and is widely used for its ability to differentiate between fears related to the process of dying versus the state of being dead, both for oneself and others.

The Death Anxiety Scale is a 15-item true/false questionnaire developed by Templer (1970) in order to assess an individual’s fear and apprehension about death (e.g. “I am very much afraid to die”). It demonstrates good psychometric properties, including strong internal consistency and test–retest reliability.

The Death Attitude Profile–Revised (DAP-R) is a 32-item Likert-scale instrument designed to assess multiple dimensions of individuals’ attitudes toward death (Wong et al., 1994). It evaluates five factors: Fear of Death, Death Avoidance, Approach Acceptance (a positive, often spiritual view of death), Escape Acceptance (viewing death as a release from suffering), and Neutral Acceptance (recognizing death as a natural part of life). The DAP-R is a revision of the original DAP, refined through factor analyses across age groups to improve conceptual clarity and psychometric properties. It has shown good to very good internal consistency and test–retest reliability as well as construct validity (Wong et al., 1994).

The Life Attitude Profile–Revised (LAP-R) is a multidimensional measure designed to assess global meaning in life, including existential attitudes relevant to death anxiety (Griffiths et al., 2016). Among breast cancer patients, the LAP-R demonstrated good psychometric properties, with factor analysis revealing four core dimensions: purpose-coherence-vacuum, choice, death acceptance, and goal seeking (Anagnostopoulos et al., 2011). The scale showed satisfactory internal consistency and convergent validity with related constructs such as stress, coping, and mental health. It was also sensitive to age-related differences in meaning, supporting its utility in both clinical and research settings focused on existential concerns and end-of-life perspectives.

The Mystical Experiences Questionnaire (MacLean et al., 2012) is a 30-item self-report scale developed to assess mystical-type experiences occasioned by hallucinogens such as psilocybin. It was derived from the original 43-item version through factor analysis and captures the core dimensions of classic mystical experience: unity/sacredness, positive mood, transcendence of time and space, and ineffability. The scale demonstrated strong internal consistency and construct validity with participants who endorsed having a mystical experience scoring significantly higher on all four factors (Table 1).

**Table 1.** Measures related to death anxiety.

Instrument	Construct	Questionnaire	Psychometric properties	Notable use
CLFD-R	Fear of death and dying (self and others)	28 items, 4 subscales, 6-point Likert scale	Excellent internal consistency; distinguishes between death/dying for self vs others	Widely used in death anxiety research
DAS	General fear and apprehension about death	15 true/false items	Strong internal consistency and test–retest reliability	Foundational tool in early death anxiety research
DAP-R	Death-related attitudes (fear, avoidance, types of acceptance)	32 items, 5-factor Likert scale	Good to very good reliability and construct validity across diverse populations	Useful in existential psychology and palliative care
LAP-R	Meaning in life, purpose, death acceptance, existential orientation	Multidimensional (e.g. coherence, choice, etc.)	Satisfactory internal consistency and convergent validity; sensitive to age and clinical status	Common in cancer and end-of-life research
MEQ-30	Mystical experience (e.g. unity, transcendence, ineffability)	30 items, 4 dimensions on a Likert scale	Strong internal consistency and construct validity in psychedelic research	Central tool in psychedelic outcome assessments

CLFD-R: Collett-Lester Fear of Death Scale – Revised; DAS: Death Anxiety Scale; DAP-R: Death Attitude Profile–Revised; LAP-R: Life Attitude Profile–Revised; MEQ-30: Mystical Experiences Questionnaire.

### Search strategy and study selection

A comprehensive search was conducted across multiple databases such as PsycINFO, ProQuest, and Google Scholar using combinations of terms such as psychedelics, death anxiety, death acceptance, psilocybin, LSD, DMT, and MDMA. The search was conducted in February 2025. Reference lists of relevant reviews and included studies were also screened to identify additional eligible studies. After duplicates were removed, titles and abstracts were screened for relevance, followed by a full-text review based on inclusion criteria.

### Data extraction and coding

For each study, the following information was extracted: author(s), publication year, sample size, psychedelic substance administered, outcome measure(s), time point of assessment, effect size (Cohen’s *d*), and standard error or variance of the effect size. For clinical studies, data were extracted from assessments conducted approximately 6 months post-treatment in three studies and 2 weeks post-treatment in one study (de la Salle et al., 2024), ensuring comparability across trials. Survey-based studies reported outcomes ranging from 1 month to 4 years following participants’ most recent psychedelic experience. Effect size data were extracted from eligible studies by recording means and standard deviations for relevant outcome measures in pre- and post-intervention groups. These values were used to calculate standardized mean differences (Cohen’s *d*), which were then converted to Hedges’ g to correct for small sample bias and allow for meaningful comparison across studies. When a study reported both death anxiety and death acceptance from the same sample, only death anxiety was included to avoid duplication of sample sizes. A sensitivity analysis was conducted, substituting the alternative measure (death acceptance), and yielded similar results. In order to make effect sizes comparable across studies assessing either death anxiety or death acceptance, all effect sizes were coded such that a positive value reflected a favorable psychological outcome (i.e. either a decrease in death anxiety or an increase in death acceptance). To achieve this, effect sizes for studies reporting reductions in death anxiety were multiplied by −1 to align the directionality with those reporting increases in death acceptance. This allowed all effect sizes to be interpreted uniformly, where higher values indicate greater psychological benefit.^1^ Substances were categorized into groups (e.g. psilocybin, LSD, MDMA) for moderator analysis. Substances represented by only a single sample were excluded from moderator analysis due to insufficient data.

### Statistical analysis

Meta-analytic computations were performed in R (R Core Team 2022) and Jamovi using the MAJOR package (The jamovi project, 2023). A random-effects model was selected for all analyses to account for heterogeneity in study design, sample characteristics, and outcome measures, and used to estimate the overall effect size of psychedelic substances on death-related outcomes. Heterogeneity was assessed using τ^2^, I^2^, and H^2^. The primary meta-analysis estimated the overall effect of psychedelic substances on death-related psychological outcomes, with standardized mean differences (Hedge’s *g*) used as the effect size metric. To explore potential differences in effect sizes across populations and contexts, two additional meta-analyses were conducted separately for studies using clinical samples and those using survey-based samples of the general population.

Two meta-regression models were also conducted. For the first meta-regression, substance type was entered as a categorical moderator to assess whether therapeutic effects varied between psychedelics. In the second, mystical experience scores were entered as a continuous moderator using only the subset of studies that reported this variable. This analysis examined whether higher reported mystical experience was associated with larger reductions in death anxiety, the dependent variable. Across all models, standardized mean differences were used as the effect size metric. Significance testing was conducted using z-values for model coefficients and Q-tests for between-group differences in moderator models. Ninety-five percent confidence intervals (CIs) were reported for all estimates. Funnel plots and Egger’s regression test were used to assess potential publication bias.

## Meta analytic results (Part I)

### Characteristics of the included studies

**Figure 1.**
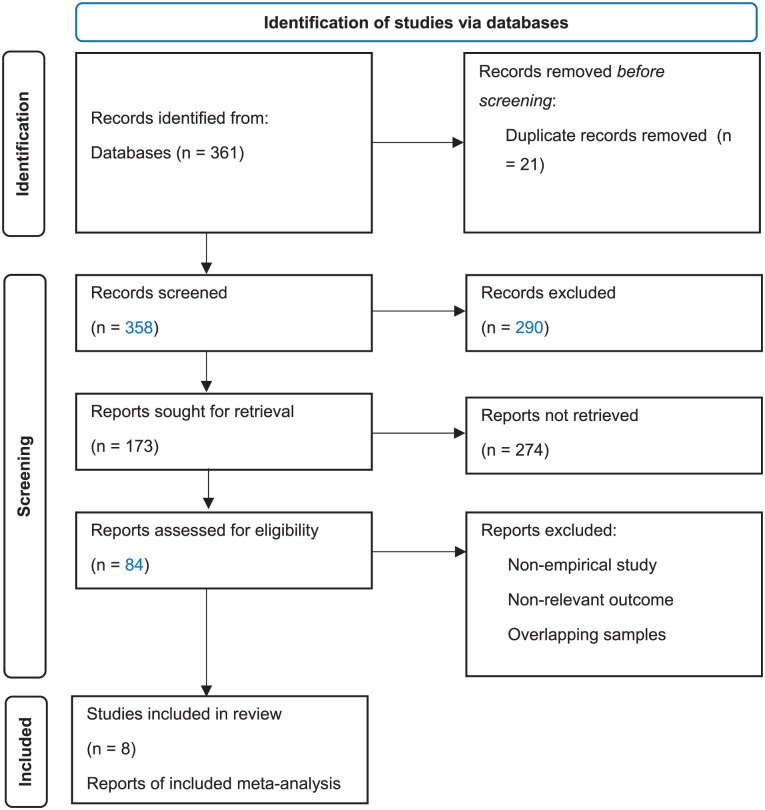
Flow diagram of article search results.

The meta-analysis included a total of 8 studies, with 3050 participants in total (Table 2). Of these, 4 were clinical trials involving treatment interventions with a combined sample size of 86 participants. The remaining four studies were survey-based and conducted with general population samples, accounting for the majority of participants. All studies included in the meta-analysis employed within-subject, pre-post designs. The four clinical-trial studies involved participants with life-threatening illnesses such as cancer. Two studies were randomized controlled trials, and two were prospective longitudinal studies conducted in naturalistic or semi-controlled settings. Sample sizes for individual studies ranged from 8 to 51 participants. One of the included studies was a long-term follow-up of participants from a prior randomized controlled trial comparing psilocybin with niacin in cancer patients (Agin-Liebes et al., 2020). Fifteen participants completed follow-up assessments 3.2–4.5 years after the initial session. To avoid duplicate data, the original study by Ross et al. (2016) was excluded from the analysis. Follow-up assessments included in the meta-analysis vary from immediate post-session to 6 months after treatment (Figure 1).

**Table 2.** Study characteristics.

Study	Participants	Substance	Measure	Research methods	Key findings
Psilocybin produces substantial and sustained decreases in depression and anxiety in patients with life-threatening cancer (Griffiths et al., 2016)	Cancer patients (*n* = 46)	Psilocybin	LAP-R Death Acceptance	Randomized, double-blind crossover trial comparing low- and high-dose psilocybin sessions in cancer patients	The high-dose psilocybin condition produced significant decreases in depression and anxiety, including death anxiety, in patients with life-threatening cancer diagnoses
Comparison of psychedelic and near-death experiences in changing attitudes about death and dying (Sweeney et al., 2022)	Individuals who reported an experience that altered their beliefs about death (*n* = 2259)	Psilocybin, LSD, Ayahuasca, DMT	DAP – Fear of Death	Observational, self-report, retrospective	-All groups reported reduced fear of death and high ratings of personal meaning, spiritual significance, and lasting psychological insight -Psychedelic groups scored higher on standardized measures of mystical and near-death experiences than non-drug group -Ayahuasca and DMT users reported stronger and more positive lasting effects than psilocybin and LSD users, which were similar to each other
Investigating the relationship between changes in metaphysical beliefs and death anxiety following a significant psychedelic experience (Moreton et al., 2024)	Individuals who reported at least one significant psychedelic experience that they felt altered their attitudes or anxieties about death (*n* = 192)	Psilocybin, LSD, Ayahuasca, DMT, Mescaline	DAP-R – Fear of Death	Observational, self-report, retrospective	-Some participants reported an increase in death anxiety, but there was an overall significant reduction in death anxiety from before to after
Long-term follow-up of psilocybin-assisted psychotherapy for psychiatric and existential distress in patients with life-threatening cancer (Agin-Liebes et al., 2020)	Cancer patients (*n* = 15)	Psilocybin	DAS	Long-term follow-up, original study was double-blind, placebo, crossover, psychotherapy alongside psilocybin administration	- Reductions in anxiety, depression, hopelessness, demoralization, and death anxiety were sustained at the first and second follow-ups - Participants overwhelmingly attributed positive life changes to the psilocybin-assisted therapy experience and rated it among the most personally meaningful and spiritually significant experiences of their lives
Longitudinal experiences of Canadians receiving compassionate access to psilocybin-assisted psychotherapy (de la Salle et al., 2024)	Cancer patients (*n* = 8)	Psilocybin	DAP-R – Fear of Death	Psilocybin-assisted psychotherapy post-treatment survey	-Significant improvements in anxiety and depression symptoms, pain, quality of life, and spiritual well-being -Attitudes towards death and desire for hastened death remained unchanged -One participant reported substantial decrease in well-being following treatment
MDMA-assisted psychotherapy for treatment of anxiety and other psychological distress related to life-threatening illnesses (Wolfson et al., 2020)	People with life-threatening illness (*n* = 17)	MDMA	DAP – Fear of Death	Randomized double-blind clinical trial combined with psychotherapy	-Overall reduction in anxiety related to life-threatening illness -Reduction in death anxiety was not significant
Reduced death anxiety and obsessive beliefs as mediators of the therapeutic effects of psychedelics on obsessive compulsive disorder symptomology (Moreton et al., 2023)	Individuals who have had a “meaningful” psychedelic experience (*n* = 312)	Psilocybin, LSD, DMT, Ayahuasca, Mescaline	CLFD-R	Observational, self-report, retrospective	-Acute subjective effects significantly predicted self-reported reductions in obsessive beliefs, death anxiety, and obsessions and compulsions following a psychedelic experience -Mediation analyses evidenced significant indirect effects of mystical experiences on obsessions and compulsions through reduced death anxiety and obsessive beliefs
Reduced death anxiety as a mediator of the relationship between acute subjective effects of psychedelics and improved subjective well-being (Moreton et al., 2023)	Individuals who have had a “meaningful” psychedelic experience (*n* = 201)	Psilocybin, LSD, DMT, Ayahuasca, Mescaline	DAP-R – Fear of Death	Observational, self-report, retrospective	-Reductions in death anxiety significantly mediated the effects of mystical experience on satisfaction with life, positive affect, and negative affect -Some of the benefits of psychedelic-induced mystical experiences on subjective well-being may emerge due to reductions in death anxiety

CLFD-R: Collett-Lester Fear of Death Scale – Revised; DAS: Death Anxiety Scale; DAP-R: Death Attitude Profile–Revised; LAP-R: Life Attitude Profile–Revised; MEQ-30: Mystical Experiences Questionnaire; LSD: Lysergic acid diethylamide; DMT: Dimethyltryptamine; MMDA: 3,4-methylenedioxymethamphetamine.

Four studies employed cross-sectional or retrospective survey designs with participants from the general population who had psychedelic experiences. Sample sizes ranged widely, from 155 to 2259, with a combined total of 2726 participants across all survey-based studies. Data collection occurred through self-report measures assessing past psychedelic experiences, death anxiety, and related constructs. Post-psychedelic death anxiety scores in the survey studies were included for individuals who had used psychedelics within the past 4 years.

Across the included studies, psychedelics were generally associated with reductions in death anxiety. While findings were largely positive, a few studies noted neutral or mixed effects, including one case where post-treatment well-being decreased (de la Salle et al., 2024). Overall, the evidence supports a potential therapeutic role of psychedelics in addressing death-related distress, particularly in clinical and palliative contexts.

### Risk of bias

**Figure 2.**
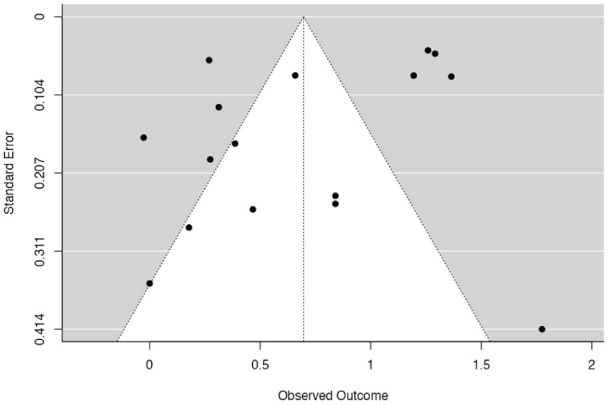
Funnel plot.

**Figure 3.**
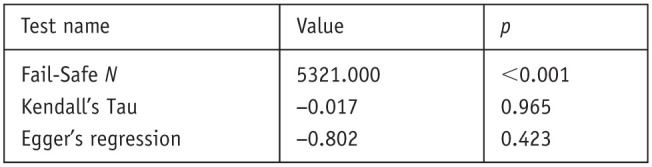
Publication bias assessment. *Note.* Fail-safe *N* calculation using the Rosenthal approach.

A funnel plot (Figure 2) was visually inspected to assess potential publication bias. The plot displayed mild asymmetry, with fewer studies appearing in the lower-left region, suggesting that smaller studies with null or negative effects may be underrepresented. This may indicate the presence of publication bias or small-study effects. To further assess potential publication bias, several statistical tests were conducted and are summarized in Figure 3. The Fail-Safe *N* was 5321 (*p* < 0.001), indicating that a large number of null-result studies would be needed to nullify the observed effect. Kendall’s Tau was −0.017 (*p* = 0.965), and Egger’s Regression intercept was −0.802 (*p* = 0.423), both suggesting no significant asymmetry in the funnel plot. These results indicate that while visual inspection of the funnel plot suggested mild asymmetry, statistical tests did not provide strong evidence of publication bias or small-study effects in the included studies.

### Main result

**Figure 4.**
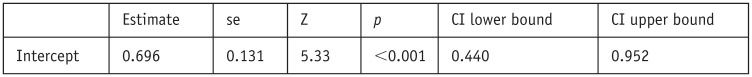
Random-effects model main result (*k* = 16). *Note.* Tau^2^ Estimator: Restricted Maximum-Likelihood. CI: Confidence interval.

**Figure 5.**
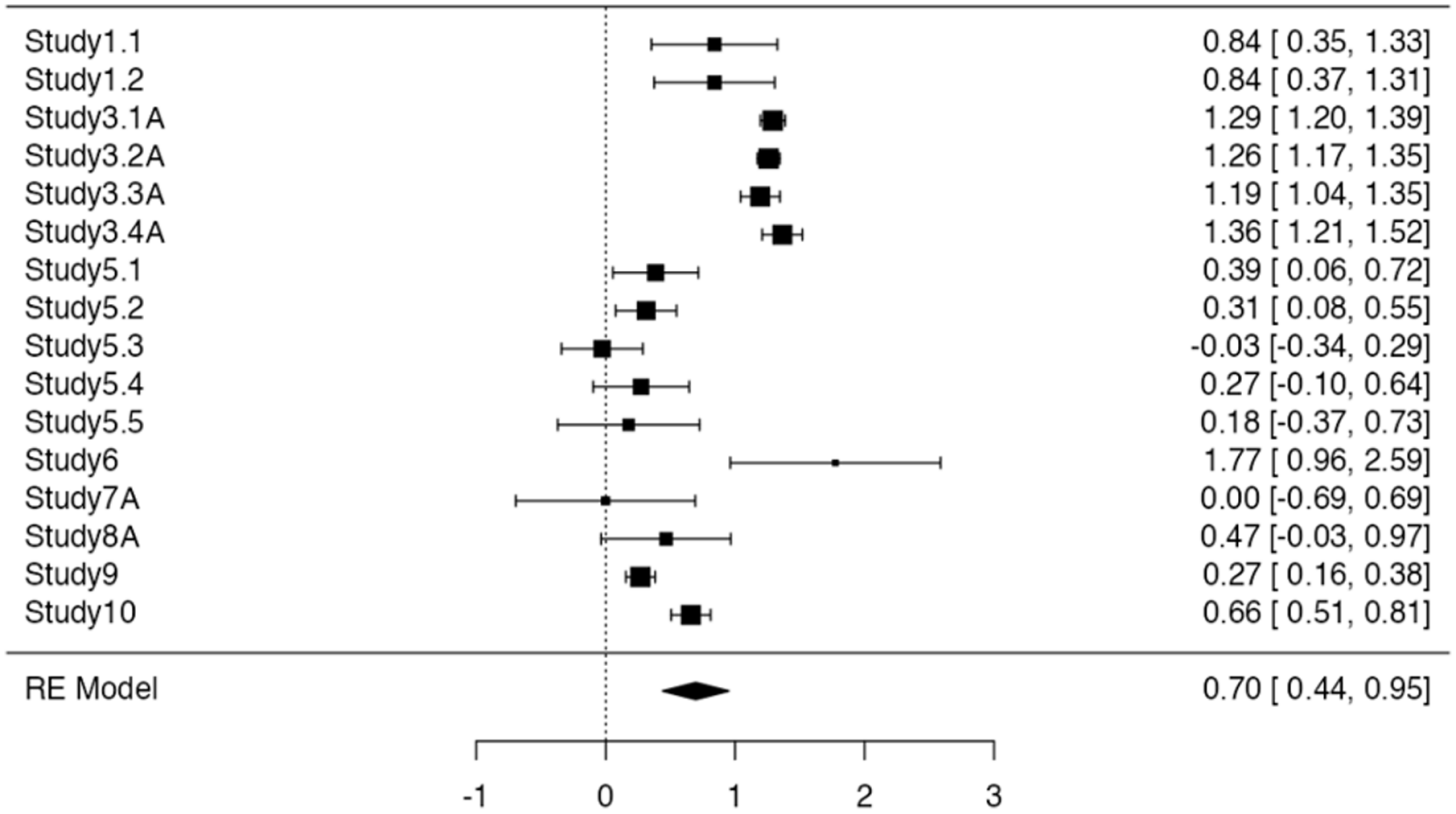
Forest plot.

A random-effects meta-analysis was conducted on all eligible studies (*k* = 16, total *n* = 8) examining the effect of psychedelics on death anxiety (Figure 4). The overall effect size was 0.696 (Standard error (SE) = 0.131, 95%CI [0.44, 0.95], *z* = 5.33, *p* < 0.001). Heterogeneity was substantial, with τ^2^ = 0.24 (SE = 0.099), I^2^ = 96.3%. The corresponding forest plot is shown in Figure 5.

### Subgroup analyses clinical and general population

**Figure 6.**
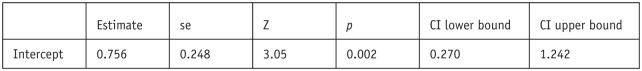
Random-effects model clinical population (*k* = 5). *Note*. Tau^2^ Estimator: Restricted Maximum-Likelihood. CI: Confidence interval.

A separate analysis was conducted on studies using clinical samples (*k* = 5, total *n* = 4). The effect size was 0.756 (SE = 0.248, 95%CI [0.27, 1.24], *z* = 3.05, *p* = 0.002) (Figure 6). Heterogeneity was τ^2^ = 0.22 (SE = 0.22), I^2^ = 72.6. The longitudinal study by Agin-Liebes et al. (2020) evaluated death anxiety at multiple time points, including immediately post-treatment, 6.5–8 months post-treatment, 3.2 years post-treatment, and 4.5 years post-treatment. Data was extracted from the 6.5- to 8-month time point to ensure comparability with other included clinical studies. Notably, the longitudinal data from Agin-Liebes et al. (2020) demonstrated a further reduction in death anxiety at 4.5 years post-treatment, with a substantial effect size (Hedges’ *g* = 2.73, *p* < 0.001, *n* = 14). Although the additional decrease from the 6.5–8-month time point to 4.5 years was not statistically significant (Hedges’ *g* = 0.40, *p* = 0.14), the lower mean score at the later time point suggests the potential for sustained or incrementally enhanced benefits over an extended period.

**Figure 7.**
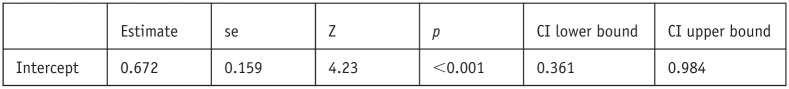
Random-effects model general population (*k* = 11). *Note*. Tau^2^ Estimator: Restricted Maximum-Likelihood. CI: Confidence interval.

For the subset of survey-based studies in the general population (*k* = 11, total *n* = 4), the effect size was 0.67 (SE = 0.16, 95%CI [0.36, 0.98], *z* = 4.23, *p* < 0.001) (Figure 7). Heterogeneity was τ^2^ = 0.26 (SE = 0.12), I^2^ = 97.63.

### Moderator analyses substance and mystical experience

**Figure 8.**
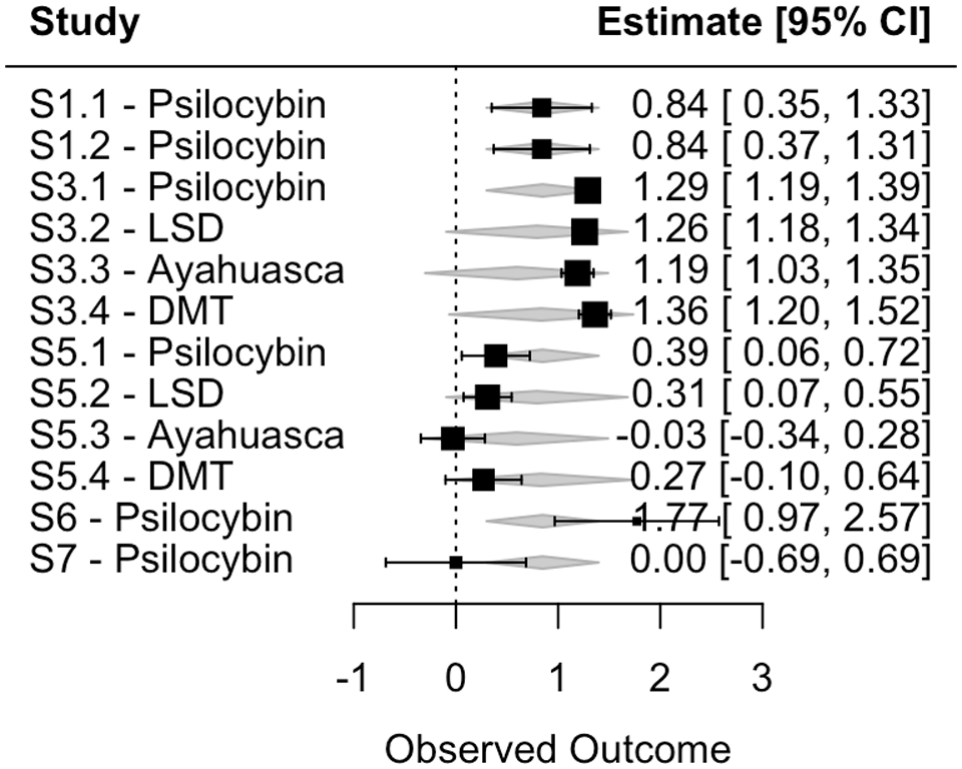
Forest plot psychedelic substance.

Moderator analyses were conducted using mixed-effects meta-regression to examine whether age (*k* = 15), gender (% female, *k* = 15), or the type of psychedelic substance (k = 2) moderated the effect on death anxiety. Substances with only one sample were excluded. Mean age was not a significant moderator (β = 0.005, SE = 0.013, *p* = 0.688), nor was gender (β = −0.004, SE = 0.008, *p* = 0.632). CIs for both moderators included zero. Substance type was also not a significant moderator, QM(df = 3) = 0.24, *p* = 0.97, indicating no significant differences between substances in their effects. However, interpretation is limited by the small and uneven distribution of studies across substance types. The forest plot illustrating the moderator results for substance type can be seen in Figure 8.

**Figure 9.**
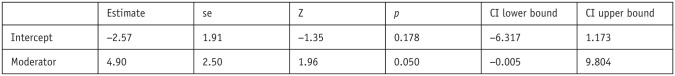
Mixed-effects model mystical experience (*k* = 5). *Note*. Tau^2^ Estimator: Restricted Maximum-Likelihood. CI: Confidence interval.

A mixed-effects meta-regression model was conducted to examine whether the level of mystical experience moderated the effect of psychedelic use on death anxiety (*k* = 5) (Figure 9). The moderator was statistically significant (*B* = 4.90, SE = 2.50, *z* = 1.96, *p* = 0.050, 95%CI [−0.005, 9.804]), indicating that higher mystical experience scores were associated with larger reductions in death anxiety. The intercept was not statistically significant (*B* = −2.57, SE = 1.91, *z* = −1.35, *p* = 0.178, 95%CI [−6.317, 1.173]).

## Systematic review results (Part II)

Studies that could not be statistically analyzed due to insufficient quantitative data were analyzed in narrative synthesis, in accordance with PRISMA guidelines.

The systematic review included ten studies with a combined total sample size of 2683 participants (Table 3). Study designs varied and included qualitative interviews, cross-sectional surveys, and case studies. Psychedelics examined across studies included LSD, MDMA, DMT, psilocybin, ayahuasca, ketamine, and unspecified psychedelic substances. Participants ranged from individuals with life-threatening illnesses to general population samples and self-identified psychedelic users. Data collection methods included semi-structured interviews, case reports, and self-report surveys, with a primary focus on subjective experiences, existential or spiritual themes, and psychological processes associated with psychedelic use in the context of death or death anxiety.

**Table 3.** Study characteristics.

Study	Participants	Substance	Research methods	Key findings
A qualitative analysis of MDMA-assisted therapy for anxiety associated with life-threatening illness (Barone et al., 2022)	Adults with a LTI who took part in clinical trials of MDMA on anxiety (*n* = 6)	MDMA	Qualitative semi-structured interviews after completion of MDMA clinical trial	-increased ability to cope with LTI -reduced psychological symptoms -improved vitality and quality of life -feeling more resourced -reconnection to life and greater emotional resilience in response to trauma and medical relapse
Patient experiences of psilocybin-assisted psychotherapy (Belser et al., 2017)	Adults with clinically elevated anxiety associated with cancer diagnosis (*n* = 13)	Psilocybin	Qualitative interviews using interpretative phenomenological analysis, participants received a moderate dose of psilocybin and psychotherapy	-relational embeddedness -emotional range -meaningful visual phenomena -revised life priorities -exalted feelings of joy, bliss, and love -a movement from feelings of separateness to interconnectedness -experiences of transient psychological distress
Ayahuasca-induced personal death experiences (David et al., 2023)	Ayahuasca users with multiple experiences (*n* = 54)	Ayahuasca	Quantitative measures including DAS, questionnaires	-ayahuasca personal death experiences (APD) occur in 50% of users and are associated with an increased sense of transcending death -DAS scores did not differ whether or not participants had an APD experience -subjective experiences during psychedelic sessions may influence attitudes toward death
Embracing change: Impermanence acceptance mediates differences in death processing between long-term ayahuasca users and non-users (David et al., 2025)	Ayahuasca users with multiple experiences (*n* = 54) and non-users (control group) (*n* = 53)	Ayahuasca	Cross-sectional design using self-report questionnaires and behavioral assessments measuring death anxiety, including the DAS and LAP-R	-ayahuasca users showed significantly lower death anxiety and greater death acceptance compared to non-users -these effects were mediated by impermanence acceptance rather than demographic or personality factors. -within the ayahuasca group, lifetime ego dissolution predicted greater impermanence acceptance.
Pahnke’s Good Friday Experiment: A long-term follow-up and methodological critique (Doblin, 1991)	White male Protestant divinity students (*n* = 16)	Psilocybin	Qualitative and quantitative long-term follow-up study involving personal interviews conducted 25 years after the initial experiment	-mystical experience was reported as being associated with a reduction in death anxiety -experiences of death/dying were described as being associated with reduced fear of death -several participants reported experiences that led to reductions in their fear of death and changes in their perceptions of what death is
Psychedelic-assisted therapy for palliative care within a home treatment setting: A case report (Federico et al., 2024)	Case study on a Swiss patient with advanced throat cancer (*n* = 1)	LSD	Home treatment of psychedelic-assisted therapy, qualitative analysis, 35-minute post-treatment feedback session, and continued follow-ups with a psychiatrist for up to 6 months	-home treatment of psychedelic therapy is a feasible option that can increase patient comfort and reduce costs -patient presented with more energy and a better mood post-session -patient reported feelings of ecstasy and peace afterwards, reduction of existential distress, and less focus on his illness
Death anxiety among users and non-users of psychedelics (Garcia et al., 2025)	Brazilian adults (*n* = 517)	LSD, ayahuasca, DMT, psilocybin, mescaline, ibogaine, cannabis, MDMA, or ketamine.	Quantitative measures including DAS, online questionnaires	-participants who have never used psychedelics had increased death anxiety -there is a negative relationship between death anxiety and mystical/religious factors of death transcendence -the possibility of transcending death is what reduces death anxiety, which psychedelics can facilitate
Ketamine-enhanced psychotherapy: preliminary clinical observations on its effects in treating death anxiety (Kolp et al., 2007)	Two end-stage cancer patients	Ketamine	Two case studies, five sessions of ketamine-enhanced psychotherapy, qualitative interviews	-one participant had a positive experience with the treatment and reported a significant decrease in death anxiety -the second participant had a negative experience and noted an increased fear of “nonexistence” -the negative experience was partially attributed to extensive past use of psychedelic drugs
The ritual use of ayahuasca during treatment of severe physical illnesses (Maia et al., 2021)	Adults who participated in an ayahuasca ceremony after diagnosis of a severe or life-threatening illness (*n* = 14)	Ayahuasca	Semi-structured qualitative interviews with open-ended questions	-ayahuasca can facilitate illness acceptance through psychological mechanisms such as introspection, catharsis, and perspective-changing -ayahuasca experiences led participants to reflect on death perceptions and commonly reduced death-related fear and distress
Differences in existential perspectives as a function of having a MTE (Sielaff et al., 2023)	Study 1: undergraduate students (*n* = 867) Study 2: (*n* = 1086) (replication of study 1)	Any psychedelic substance	Two cross-sectional studies, quantitative measures including DAP, online survey	-reduced fear of death in participants who have had a “mystical-type experience” (MTE) -MTEs were linked with a more intrinsic and growth-oriented worldview

DAS: Death Anxiety Scale; LAP-R: Life Attitude Profile–Revised; LSD: lysergic acid diethylamide; DMT: dimethyltryptamine; MMDA: 3,4-methylenedioxymethamphetamine; LTI: Life-threatening illness; MTE: Mystical-type experience.

### Narrative synthesis

In order to conduct the narrative synthesis, the included studies were grouped according to recurring psychological themes that emerged across studies. Emergent themes included emotional processing, mystical-type experiences, death transcendence, and ontological beliefs.

### Emotional processing in life-threatening illness

The studies included in the systematic review collectively suggest that psychedelic experiences may reduce death anxiety and enhance existential well-being, though the nature and impact of these experiences vary. Qualitative studies of MDMA and psilocybin-assisted psychotherapy in individuals with life-threatening illnesses reported increased coping, vitality, and emotional resilience, alongside deep emotional and relational shifts such as greater interconnectedness and revised life priorities (Barone et al., 2022; Belser et al., 2017).

While the clinical intention of the treatment was to address anxiety around life-threatening illness, several participants found themselves exploring broader unresolved trauma and emotional wounds. Rather than focusing exclusively on death, the therapy facilitated a form of emotional processing, which may have indirectly improved death attitudes through greater integration and emotional peace. One clinical case study on psychedelic-assisted therapy delivered in a home setting was carried out for a patient with advanced throat cancer experiencing significant existential distress (Federico et al., 2024). The intervention was associated with reductions in distress related to the illness, alongside improvements in mood and psychological well-being. The patient reported feelings of ecstasy and peace, reduced existential suffering, and an emotional shift toward his illness and mortality following treatment.

Several participants described a shift from death-related despair to a more peaceful, accepting attitude toward mortality after participating in an ayahuasca ceremony (Maia et al., 2021). The experience of ayahuasca treatment appeared to amplify the reflective processes already initiated by their illness. One participant with cancer stated: *“I don’t mean I want to die, I don’t want to, I want to live long, but I don’t see death with that fear I saw it first, with uncertainty, despair” (Maia et al., 2021: 276).* This reflects the recurring theme in the qualitative literature that death acceptance does not necessarily mean embracing death but rather coming to terms with its inevitability in a way that reduces distress and increases psychological well-being.

### Being in the present moment

Another common theme was the feeling of being in the present moment. Several individuals described a stronger ability to stay anchored in the present, rather than ruminating on past trauma or future fears such as cancer recurrence or death (Barone et al., 2022). This shift appears to buffer against anxiety by promoting psychological flexibility and self-compassion:

*You know, it changed the way I am in the world and the way I orient myself from the thing that had happened and the things that might happen. Cancer or no cancer, that is such a gift. To really be able to be in the present moment is just such a gift.* (p. 13)

### Mystical-type experiences, death transcendence, and ontological beliefs

Studies further support the role of mystical-type experiences in shaping death attitudes. For example, users who had experienced such states (Sielaff et al., 2023) or had ongoing psychedelic use (Garcia et al., 2025) reported less fear of death and a greater sense of death transcendence. However, findings were not universally positive, and one case study (Kolp et al., 2007) reported increased fear of nonexistence in a participant with a history of extensive psychedelic use. Overall, the evidence suggests that the subjective quality of psychedelic experiences may be central to their effects on death anxiety.

In Garcia et al.’s (2025) large cross-sectional study, individuals who had never or rarely used psychedelics reported higher death anxiety compared to those with more recent psychedelic use. Aspects of death transcendence, and specifically the Creative and Religious factors of the Death Transcendence Scale, emerged as consistent predictors of lower death anxiety across regression models, while mystical and religious experiences were negatively correlated with death anxiety. These findings suggest that psychedelics may facilitate experiences in which participants perceive existence as continuing beyond physical death rather than directly reducing death anxiety.

Participants in Sielaff et al.’s (2023) study who had previously experienced a mystical-type experience reported greater belief in death as a passage and higher levels of intrinsic spirituality and personal growth, as well as a lower fear of death, compared with those who had not. These effects were often reported outside of a therapeutic context, suggesting that mystical-type experiences are associated with shifts in worldview and reduced death anxiety even in non-clinical settings.

Many participants in the study by Barone et al. (2022) described experiencing mystical-type states, such as feelings of interconnectedness, timelessness, and spiritual transcendence. Unlike the more dissociative or ego-dissolving states often seen with psychedelics, participants reported that their MDMA-induced mystical experiences remained grounded and self-connected.

In the qualitative study by Belser et al. (2017), participants undergoing psilocybin-assisted therapy for cancer-related distress described a range of emotionally intense and spiritually significant experiences that appeared to play a central role in reshaping their attitudes toward death. Common themes included profound emotional release, dissolution of ego boundaries, and a sense of interconnectedness with others, nature, or the universe. For example, several participants reported powerful moments of reunion with deceased loved ones or guiding spirits, often described as deeply comforting and affirming. One participant recalled, *“I was flying through space with the spirit guide, and I encountered three people who are dead who were very close to me. . . and they all gave me reassuring messages in space”* (Belser et al., 2017: 366). These encounters with symbolic figures of death and the afterlife offered reassurance, closure, and a feeling of peace.

The dissolution of a fixed self was reported by 9 out of 13 participants and was often accompanied by a transcendent awareness of unity: *“We’re all going to be connected again in the universe”* (Belser et al., 2017: 369). Similarly, a participant in the follow-up study conducted by Doblin (1991) expressed a feeling of continuation of consciousness after death:

*I felt that I was caught up in the vastness of Creation. . . I did experience that kind of classic kind of blending. . .. The main thing about it was a sense of timelessness. Death looked different. I got the impression, the sensation. . . that what people are essentially in their essence that somehow they would continue to live. They may die in one sense, the physical sense, but their being in heaven would survive. (p.16).*

This perspective, in which life and death are seen as part of a continuous whole, reinforces findings in other studies where shifts in beliefs and mystical-type experiences were predictive of reduced death anxiety (Griffiths et al., 2016; Ross et al., 2016). Some participants in Belser et al.’s (2017) study experienced symbolic healing, including visions of cancer being ejected from their bodies or being lovingly accepted as part of their embodied reality, suggesting that death acceptance may also arise through a deeper acceptance of life as it is.

### Personal death experiences and psychological distress

David et al. (2023) investigated a common phenomenon reported in ayahuasca ceremonies known as the Ayahuasca-induced Personal Death (APD) experience. These experiences, which involve a sensation of dying or ego death, were reported in at least half of the participants who consumed ayahuasca and were reported to deeply impact participants’ attitudes toward death. Notably, participants often perceived the experiences as highly intense, and many described lasting effects on their sense of the continuity of the soul/consciousness after death. However, despite their existential and transformative nature, APDs were not associated with reduced death anxiety.

Building on this work, David et al. (2025) conducted an analysis of death processing and its underlying mechanisms in ayahuasca users compared to non-users. Ayahuasca users exhibited lower death anxiety, fear of death, and death-avoidant behaviors, alongside greater death acceptance. Mediation analyses demonstrated that these differences were specifically mediated by *impermanence acceptance*.

While many participants across studies reported decreased death anxiety and increased peace through mystical or spiritually significant experiences, not all psychedelic encounters resulted in positive transformations. In a case study by Kolp et al. (2007), one participant described an experience that starkly contrasted with the common themes of unity, transcendence, and acceptance. Instead of healing, he encountered overwhelming existential terror, loss of self, and a deepened fear of nonexistence:

*As soon as the drug started working, my mind separated from my body and, in turn, started going into oblivion. I realized I am dying and a strong fear of nonexistence completely overwhelmed me. My mind was finally gone and I was sucked into an infinite ocean of unconditional sorrow. Some part of me – the one of an observer – continued existing; however, it began decaying as well. I died as a human many times, each time from different causes, somehow re-incarnating again, each time regressing on a lower level. I then started dying and re-incarnating as a mammal, again regressing from higher forms to lower ones, next as a bird, after that as a fish, and so on, until I became a primordial protoplasm, at which point I blacked out. Only when the drug stopped working did I recognize that I was still alive. It was the worst bummer (a bad ‘trip’) I’ve ever had.* (p. 10).

Following this experience, the participant stated that he “*now knows for sure his mom was right when she taught there is no life after death.*” Rather than reducing fear, this session appeared to instill a nihilistic worldview. The researchers noted that this individual’s extensive prior psychedelic use may have played a role in the lack of therapeutic effect, suggesting that prior expectations or neurochemical habituation could potentially alter the impact of psychedelic therapy. Reports of difficult psychological experiences serve as a reminder that psychedelic experiences are not uniformly beneficial and can, in some cases, intensify death anxiety or existential dread if the individual’s mindset or psychological vulnerability is not adequately addressed (Evans et al., 2023; Kolp et al., 2007).

In Doblin’s (1991) follow-up study, only two psilocybin subjects reported having experiences that were completely positive without significant psychological struggles. Doblin stated that difficult moments may have been understated in the original experiment (Pahnke, 1963), with most participants reporting moments in which they feared they were dying, going crazy, or were not mentally strong enough to face what they were experiencing. One patient stated:

*For a brief moment there, I was physically dying. My insides were literally being scooped out, and it was very painful. . .. I said to myself. . . that nobody should have to go through this. . . it was excruciating to die like that. Very painful. And I died. . .. (p. 18).*

However, the struggles were all resolved during the course of the study, and the subjects stated that these experiences contributed to their learning and growth. One participant reported that the subjective experience of dying may have contributed to the reduction in death anxiety, stating:

*When you get a clear vision of what [death] is and have sort of been there, and have left the self, left the body, you know, self leaving the body, or soul leaving the body, or whatever you want to call it. . . In a sense [it takes away the fear of dying]. . . because you’ve already been there. (p. 15).*

## Discussion

This review and meta-analysis provide converging evidence that psychedelic-assisted experiences are associated with meaningful reductions in death anxiety. According to Cohen’s (1988) guidelines for standardized mean differences, the overall effect size observed (*g* = 0.70) falls within the moderate to large range, suggesting that psychedelic interventions may be effective in addressing death anxiety. The consistency of findings across clinical and survey-based samples reinforces the potential generalizability of these effects, even though substantial heterogeneity indicates variability in outcomes across studies. This variation likely reflects differences in methodology, participant characteristics, and psychedelic compounds used. The findings are particularly meaningful in clinical contexts, where psychedelics appear to provide substantial support to individuals facing life-threatening illnesses. The clinical subgroup (*k* = 5) yielded a slightly higher effect size (*g* = 0.756) compared to the survey-based subgroup (*g* = 0.67), a difference of Δ = 0.09, highlighting a potential for these interventions in palliative care settings.

However, it is important to acknowledge potential adverse psychological effects when considering the potential implementation of psychedelics as a widespread treatment option. For example, one large-scale mixed-methods study by Evans et al. (2023) (*n* = 608) found that some individuals experienced prolonged existential distress and fear following psychedelic use, with 33 participants experiencing an increased fear of death/dying afterwards and a subset reporting symptoms persisting for over a year. Additionally, some studies included in this review reported neutral or mixed effects, including where overall well-being decreased after treatment (de la Salle et al., 2024; Kolp et al., 2007). However, these were exceptions to the overall trend. Notably, the MDMA-assisted psychotherapy trial (Wolfson et al., 2020) did not find a significant change in death anxiety, though it did observe reductions in general anxiety.

The results of the moderator analysis indicate that mystical-type experiences significantly moderated reductions in death anxiety, with higher levels of mystical experience associated with greater benefit. This aligns with qualitative findings from the systematic review, where participants commonly described ego dissolution, encounters with spiritual guides, and feelings of profound interconnectedness as central to their transformation. In Belser et al. (2017), for example, participants often reported intense emotional experiences, the presence of deceased loved ones, and a shift from separateness to unity; phenomena closely tied to mystical-type experiences. These experiences may help reframe death not as an isolated event, but as a return to or continuation within a broader, interconnected whole. In this way, psychedelics may provide a psychological framework that allows death to be approached with acceptance rather than fear.

In the systematic review, additional potential moderators emerged qualitatively, including acceptance of impermanence, death transcendence, and ontological beliefs. Several studies reported shifts in ontological or afterlife beliefs following psychedelic or mystical-type experiences, often toward viewing death as a passage (Garcia et al., 2025; Moreton et al., 2024; Sielaff et al., 2023). However, these belief changes were not consistently associated with reductions in death anxiety. For example, both David et al. (2023) and David et al. (2025) found that while ayahuasca users and individuals reporting personal death experiences endorsed stronger transcendence-related beliefs, ontological beliefs did not mediate differences in death anxiety or death acceptance. Instead, David et al. (2025) identified acceptance of impermanence as the key mechanism explaining reduced death anxiety.

Furthermore, Moreton et al. (2024) demonstrated that psychedelic experiences were often associated with changes in metaphysical beliefs. However, only panpsychism correlated with changes in death anxiety, and mystical experience predicted death anxiety independently of belief change. Although psychedelic experiences are frequently accompanied by shifts in ontological beliefs, such belief changes are neither necessary nor sufficient to explain reductions in death anxiety and do not consistently function as moderators. Experiential processes such as mystical-type experiences, death transcendence, and impermanence acceptance seem to more reliably account for variance in death anxiety.

Themes such as emotional catharsis, surrender, and revised life priorities further support the interpretation that psychedelics may reduce death anxiety not only through spiritual insight but also by enabling the processing of repressed grief and fostering a sense of renewed purpose. Even challenging or distressing trips appeared to play a meaningful role, with participants in several studies emerging with a greater sense of clarity and peace after confronting emotionally difficult experiences. These findings suggest that the therapeutic value of psychedelics in reducing death anxiety lies not only in the pharmacological effects of the substances but also in the nature of the experiences they produce.

While not all transformative experiences are shown to reduce death anxiety, the broader pattern across the reviewed studies suggests that mystical-type experiences do play an important role. Participants who reported profound feelings of interconnectedness, ego dissolution, or encounters with the sacred often described enduring reductions in fear of death, increased acceptance, and a sense of symbolic continuity beyond life (Belser et al., 2017; Barone et al., 2022; Maia et al., 2021; Sielaff et al., 2023). These experiences may provide a psychological framework through which mortality becomes less threatening, aligning with terror management theory by invoking symbolic immortality and reinforcing meaning. This suggests that not all transformative psychedelic experiences reduce death anxiety equally, and that how a person interprets or integrates the experience might play a more significant role than the intensity or thematic content. For instance, APDs may shift ontological beliefs without necessarily alleviating the emotional or existential fear of death.

### Limitations

Several limitations should be considered when interpreting the findings of this meta-analysis and systematic review. First, the number of studies included was relatively small, limiting the statistical power and generalizability of the results. Given the emerging nature of psychedelic research, particularly in relation to death anxiety, many existing studies did not meet the inclusion criteria due to a lack of appropriate measures or insufficient reporting of outcomes. Therefore, the final sample may not fully represent the broader body of psychedelic research.

Additionally, there was substantial methodological heterogeneity among the included studies, including differences in study design, psychedelic substances used, populations sampled, assessment time points, and outcome measures for death anxiety. This variability may have introduced noise into the effect size estimates and limited the ability to draw strong conclusions about moderators or mechanisms of change.

The moderator analysis was based on only two studies, one of which contributed four substance-based samples from a large online survey. Although the samples were treated as independent based on substance groupings, the shared origin may still limit the generalizability of the findings. Furthermore, the subgroup analysis of clinical trials was based on only 86 patients across four studies, two of which did not report significant effects on death anxiety. This small sample size, coupled with mixed findings, limits the strength of conclusions that can be drawn from the clinical trial data.

Risk of bias was present in several studies, particularly in areas such as participant selection, outcome measurement, and lack of blinding or control groups. While efforts were made to assess and account for bias, these factors may have influenced the observed effects. Several of the included studies recruited participants based on their report of a meaningful psychedelic experience that influenced their attitudes toward death. This introduces a sampling bias, as individuals who experienced no change may be underrepresented. Consequently, the results may overestimate the positive effects of psychedelics on death anxiety. Although some studies did report cases of worsened anxiety, people with neutral or unchanged attitudes toward death may have been left out of the recruitment process, limiting the generalizability of the findings.

Publication bias remains a concern as well, which could indicate that studies reporting null or negative effects may be underrepresented in the literature. While this synthesis contributes to the growing understanding of psychedelics and their potential impact on death-related constructs, there is a need for more rigorous, high-quality research in this area, and the findings should be interpreted with caution.

### Practical implications

In palliative care and end-of-life contexts, the results of this review support ongoing efforts to integrate psychedelic therapies as a means of alleviating death anxiety. The effect was also present in general population samples, suggesting that psychedelics could be useful for addressing broader existential concerns that affect many individuals outside of the context of terminal illness, such as fear of death or loss of meaning.

Future research should aim to clarify the mechanisms by which psychedelic experiences reduce death anxiety. This includes investigating the role of mystical-type experiences, ego dissolution, and changes in personal values or death-related beliefs. Longitudinal studies are also needed to assess the durability of these effects, particularly in general populations where the psychedelic experience may be less structured. Additionally, the field would benefit from more rigorous, high-powered randomized controlled trials and qualitative studies exploring individual narratives of psychedelic experiences.

## Conclusion

In conclusion, this meta-analysis and systematic review support the growing body of evidence that psychedelic-assisted therapy has the potential to significantly reduce death anxiety, particularly in individuals with life-threatening illnesses. While further research is needed to clarify mechanisms and optimize protocols, the current findings suggest that psychedelics may help individuals approach the end of life with greater peace, purpose, and connectedness.

## Notes

1. In the study by Sweeney et al. (2022), individuals who have had near-death experiences were included as participants; however, for the purposes of this meta-analysis these participants were excluded from data extraction. Only the participants who had a psychedelic experience were included, bringing the initial sample size of the study from *n* = 3192 to *n* = 2259.
